# Automated Masking of AFLP Markers Improves Reliability of Phylogenetic Analyses

**DOI:** 10.1371/journal.pone.0049119

**Published:** 2012-11-09

**Authors:** Patrick Kück, Carola Greve, Bernhard Misof, France Gimnich

**Affiliations:** Zoologisches Forschungsmuseum Alexander Koenig, Bonn, Germany; J. Craig Venter Institute, United States of America

## Abstract

The amplified fragment length polymorphisms (AFLP) method has become an attractive tool in phylogenetics due to the ease with which large numbers of characters can be generated. In contrast to sequence-based phylogenetic approaches, AFLP data consist of anonymous multilocus markers. However, potential artificial amplifications or amplification failures of fragments contained in the AFLP data set will reduce AFLP reliability especially in phylogenetic inferences. In the present study, we introduce a new automated scoring approach, called “AMARE” (AFLP MAtrix REduction). The approach is based on replicates and makes marker selection dependent on marker reproducibility to control for scoring errors. To demonstrate the effectiveness of our approach we record error rate estimations, resolution scores, PCoA and stemminess calculations. As in general the true tree (i.e. the species phylogeny) is not known, we tested AMARE with empirical, already published AFLP data sets, and compared tree topologies of different AMARE generated character matrices to existing phylogenetic trees and/or other independent sources such as morphological and geographical data. It turns out that the selection of masked character matrices with highest resolution scores gave similar or even better phylogenetic results than the original AFLP data sets.

## Introduction

Amplified fragment length polymorphism (AFLP) data consist of sets of anonymous multilocus markers, in contrast to sequence based phylogenetic approaches. Unlike DNA sequencing, where each nucleotide can be determined with high degree of confidence, AFLPs can contain artificial amplifications or amplification failures of fragments which will reduce AFLP reliability especially in phylogenetic inferences - resulting in decreased resolution and/or support of phylogenetic trees. In this study, we address the difficulties in scoring AFLP profiles by measuring marker reproducibility. This approach does not solve the problem of homology assessment in AFLP data *per se*, but improves its reproducibility and accuracy, which further increases the reliability of phylogenetic reconstructions based on AFLP markers.

The AFLP technique [1] is a commonly used approach in evolutionary, ecological and population genetic studies [2], [3]. Due to the ease with which large numbers of characters can be generated, AFLP markers recently became a valuable tool even for genomic approaches in population genetics [3]–[5]. The combination of large numbers of characters and phylogenetic signal in many AFLP data sets [6], [7] has led to an increasing use of AFLP markers in phylogenetic analyses as well. In these studies, AFLP markers proved to be valuable characters to resolve phylogenetic relationships particularly among closely related taxa, but also at the family-level [3], [8].

In short, AFLP profiles of individuals are generated by amplifying preselected restriction digested DNA fragments and electrophoretic separation of the amplicons. Subsequently, a fundamental step in all AFLP studies is the conversion of the AFLP profiles into a binary presence-absence (1/0) character matrix. This process is called scoring and includes two major challenges:

- The definition of fragment size categories (hereafter also referred to as bins), i.e. a correct assessment of statistical variability of electrophoretic mobility of fragments, which is necessary to avoid “oversplitting” of identical alleles into separate characters or merging of non-identical alleles into one character (technical homoplasy) [9].
- The assessment of fluorescent intensity, i.e. phenotype calling based on presence or absence of fragments within each bin and for each sample, where the presence of a fragment is coded with 1 (“present” allele) and the absence of a fragment is coded as 0 (“null” allele).

Finally, the binary character matrix forms the basis for all evolutionary inferences. Scoring AFLP profiles is a highly demanding task and Bonin et al. [10] showed that the scoring process is the most error-prone step in the AFLP procedure due to the difficulty and subjectivity in correctly reading profiles. The “Holy Grail” of AFLP scoring has not been found yet and screening the literature reveals many ways - from manually and semi-automated to automated scoring – how AFLP profiles were scored [8], [11]–[25]. In most studies scoring has been performed in a semi-automated fashion by manually inspecting AFLP profiles evaluated by commercial available software packages. The recent development of several scoring scripts [9], [26]–[29], however, indicates the common need for automated scoring approaches which go beyond those provided by commercial software. In contrast to the widespread practice of manual and semi-automated scoring, automated scoring can be objective (regarding the automatic application of user specified parameter settings), reproducible and far less time-consuming.

Arrigo et al. [9], for example, proposed an automated approach (RawGeno) which focuses on the definition of bin width and analyzed the influence of oversplitting of identical alleles or technical homoplasy of non-identical alleles on estimates of genetic diversity and genetic structure. They showed that wrong definitions resulted in a loss of discriminatory power and decrease the robustness of results in population genetic and phylogeographic analyses. Evaluating several error rate estimates [10], [30] as selection criteria for bin width, Arrigo et al. [9] introduced the information content per bin (I_bin_) as a new and valuable optimality criterion.

Whitlock et al. [26] and Herrmann et al. [28] developed two alternative marker selection scripts (AFLPScore and scanAFLP) based on fragment fluorescent intensity (peak height) thresholds. Both scripts allow reproducible selection and scoring of markers after AFLP profiles were evaluated by commercial genotyping software. Whereas Whitlock et al. [26] only used mismatch error rate estimations [10] to optimize the AFLP scoring thresholds, Herrmann et al. [28] also focus on population genetic parameters like genetic diversity and principal coordinate analyses (PCoA). Herrmann et al. [28] showed that scanAFLP reduced mismatch error rates, i.e. noise in the data, while retaining patterns of population genetic structure. Both approaches did not consider aspects of bin width definition like Arrigo et al. [9].

The relevance of these studies for phylogenetic analyses is not immediately obvious. In a phylogenetic context, Holland et al. [31] suggested analyzing AFLP profiles with commercial scoring software (GeneMapper by Applied Biosystems and GeneMarker by SoftGenetics). They showed that optimizing scoring parameters of commercial software, such as peak height, bin widths, and minimum fragment size, significantly increases the quality and resolution of the binary character matrix and resulting phylogenetic tree, respectively [3], [31]. As optimality criterion and proxy of accuracy, they used the resolution of phylogenetic trees to choose between data matrices constructed with different parameter settings. The resolution score of each resulting phylogenetic tree/character matrix is based on bootstrap values. Based on studies of Hillis and Bull [32] and Taylor and Piel [33], Holland et al. [31] assumed that a high resolution score is correlated with accuracy and indicates increasing phylogenetic information in the character matrix. Further it can be expected that both the quality and the number of characters will have an effect on accuracy and resolution. To disentangle these two effects and to get a measure of character quality independent of sequence length, Holland et al. [31] defined a normalized resolution score. These normalized resolution scores showed that most of the differences in resolution could be explained by a difference in the number of characters and that the presence of more characters leads to higher resolution. Holland et al. [31] also showed that parameter settings of commercial software, which generated data matrices with the least mismatch error rate [10], did not give superior phylogenetic resolution due to the considerable loss of valuable characters. They concluded that minimizing error rates in AFLP character matrices is a trade-off between the number of lower and high quality characters.

In the present study, we introduce a new automated scoring approach, called “AMARE“ (**A**FLP **MA**trix **RE**duction) to evaluate the reliability of each marker and to simultaneously perform error rate estimations. Compared with other scoring approaches [9], [26]–[29], AMARE focuses on replicates. AMARE thus makes marker selection dependent on marker reproducibility itself, which is the most rigorous way to control for artificial amplification errors and an objective measure of data quality.

Using commercial software packages for bin width definition and peak height detection, AMARE serves as a second filter for marker selection. In short, AMARE tries to keep as many characters as possible by inspecting the quality of replicates of individuals. Low quality replicates are discarded from the data set dependent on a replicate reliability threshold. Further, the user defines an acceptance threshold of unreproducible markers. Sizing precision, defined as the ability to obtain reproducible sizing of DNA fragments from injection to injection on a capillary instrument, is not perfect [34]. Consequently, the user can indicate a threshold of allowed distances between differently sized bins corresponding to the standard deviation of the sequencer’s sizing precision. Considering these parameters, AMARE makes a major contribution to all other currently available automated scoring approaches.

To decide between character matrices constructed with different parameter settings of AMARE, we considered the effect of marker selection on mismatch error rate estimations [10], [31], principal coordinate analyses (PCoA) [28], stemminess [35] and resolution scores [31]. As in general the true tree (i.e. the species phylogeny) is not known, we tested AMARE with empirical, already published AFLP data sets [8], [11], [31], and compared tree topologies of different AMARE generated character matrices to existing phylogenetic trees and/or other independent sources such as morphological and geographical data.

The aims of this study are (i) to show that AMARE improves the signal-to-noise ratio in AFLP binary character matrices in an objective and repeatable way by increasing the number of valuable characters and reducing background noise; and (ii) to highly encourage the AFLP community to make AFLP profiles publicly available and binary matrix generation more transparent by using fully automated scoring software.

## Methods

### Concept

The approach optimizes the signal-to-noise ratio in AFLP data sets (single or multiple primer combinations). It identifies AFLP genotyping errors as the number of unreproducibly scored markers between replicated AFLP profiles of single individuals. As a measure of overall data quality, it simultaneously estimates general replicate error rates. Strength and accuracy of the approach depend on the number of replicates and whether they are representative for the whole data set.

### Definitions

#### Bin

The AFLP profile of an individual is defined as a set of fragments characterized by their electrophoretic mobility, i.e. fragment size, and fluorescent intensity. Common genotyping software categorizes AFLP profiles into bins sorted by fragment size. A bin usually covers a size range of ∼1 base (b), in which all fragments are considered homologous and thus a single character/marker. AFLP profiles are converted into a binary character matrix by recording the presence/absence of fragments within each bin.

#### Replicates

Replicates, i.e. replicated pairs of AFLP profiles, are generated from re-amplification and re-analyses of identical DNA sources of one individual or re-extraction of a single individual/tissue. For each bin, AMARE assesses reproducibility of markers evaluating pairs of replicated AFLP profiles. AMARE transforms these replicates into pairs (*i,j*) representing the observed state 0 (fragment absence) or 1 (fragment presence) within each bin (Table 1). In Table 1, markers of bin 1, 2, 3, 4, 7, 8 and 9 are reproducible, whereas markers of bin 5 and 6 are unreproducible among replicated AFLP profiles of Individual A. For bin 10, AFLP reactions of Individual A_Replicate2_ have failed for technical reasons, therefore it got the entry ”?”. AMARE uses replicates of single individuals and not incongruences between different individuals to assess reproducibility of AFLP markers. Exclusion or inclusion of a bin in the complete data matrix is thus based only on the given set of replicates. The starting point is a matrix of *n* replicates, populated with (?,?), (0,?), (?,0), (1,?), (?,1), (0,0), (1,0), (0,1), and (1,1) pairs. The masking of bins is subsequently applied to the complete binary character matrix of all sampled individuals. In cases of more than two replicates of one individual, any further replicate is not considered by AMARE, but can be kept in the whole data set as control sample. AMARE uses three criteria (thresholds) to mask the matrix:

- Bin reliability (BR): We define N*_x_* (*i,j*) as the observed number of the pair (*i,j*) at bin*_x_* for all *n* replicates, where pair (*i,j*) is one of the nine pairs (?,?), (0,?), (?,0), (1,?), (?,1), (0,0), (1,0), (0,1), and (1,1). Bin reliability, BR*_x_*, is defined as the relative number of reproducible markers for bin*_x_* over all *n* replicates:

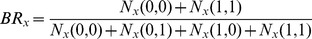
BR*_x_* will range between 0 and 1. Ambiguously scored bins, e.g. (?,?), (0,?), are not considered. A predefined threshold BR sets the acceptance value of the minimal number of reproducible (0,0) and (1,1) bin states. If bin*_x_* has a BR*_x_* below the predefined threshold BR, BR*_x_*≤BR, bin*_x_* is considered unreliable and will be masked in the matrix. If bin*_x_* contains only (0,0) pairs or has no (1,1) pairs among replicates, it will be masked as well.

- Replicate reliability (RR): Replicate reliability, RR_y_, is defined as the relative number of reproducible markers between replicates *y* of a single individual over all *n* bins. We define M_y_ (*i,j*) as the observed number of pair (*i,j*) between replicates *y* of a single individual, where the pair (*i,j*) is one of the nine pairs listed before. RR_y_ is then defined as:

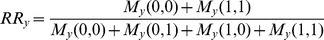
Replicates are masked if the marker reproducibility is below a certain threshold, RR_y_ ≤ RR. RR_y_ can range from 0 to 1.

- Minimum bin distance (BD): Bin distance, BD*_z_*, defined as the distance between differently sized bins. A threshold BD is set to demand a minimum distance between differently sized bins. Both bins are masked, if BD*_z_*≤BD. BD must be between 0 and 1 otherwise BD is greater than a bin width of one nucleotide.

**Table 1:** AMARE replicate transformation.

Bin	1	2	3	4	5	6	7	8	9	10
**Individual A_Replicate1_**	0	0	1	1	0	0	1	1	0	0
**Individual A_Replicate2_**	0	0	1	1	1	1	1	1	0	?
**Pairs**	(0,0)	(0,0)	(1,1)	(1,1)	(0,1)	(0,1)	(1,1)	(1,1)	(0,0)	(0,?)

This example shows how AMARE transforms replicates into pairs (*i,j*) representing the observed state 0 (fragment absence) or 1 (fragment presence) within each bin.

#### Error rate calculations

Error rates among replicates are calculated: (1) the replicate mismatch error rate r*_Bonin_* [10],
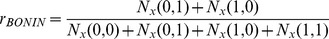
is defined as the relative number of unreproducible N(0,1) and N(0,1) summed over all *n* replicates and (2) the average *Jaccard* mismatch error rate r*_Jaccard_* [31],



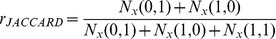
which divides the number of unreproducible N(0,1) and N(1,0) markers by the sum of reproducible N(1,1) and unreproducible N(0,1) and N(1,0) markers. The error rate reflects the quality of the replicates; the higher the error rate, the higher the proportion of unreproducible markers among replicates.

### Matrix Masking

The idea of the masking process is to mask unreliable bins within an AFLP binary character matrix in order to improve the overall reliability of the character matrix and thus minimize genotyping errors. The starting point is the assumption that a representative sample of replicates, i.e. >10% of all sampled individuals, can help to identify unreliable bins. Unreliable bins are defined as bins which show a high number of incongruent scorings among these replicates. A threshold of BR is used to identify these bins. We use a step-wise increment of the BR and RR thresholds to identify character matrices with replicate mismatch error rates (r*_Bonin_* or r*_Jaccard_*) <0.1 and a maximum number of bins. After unreliable bins have been identified among replicates they will in consequence be masked in the complete data as well.

**Figure 1:**
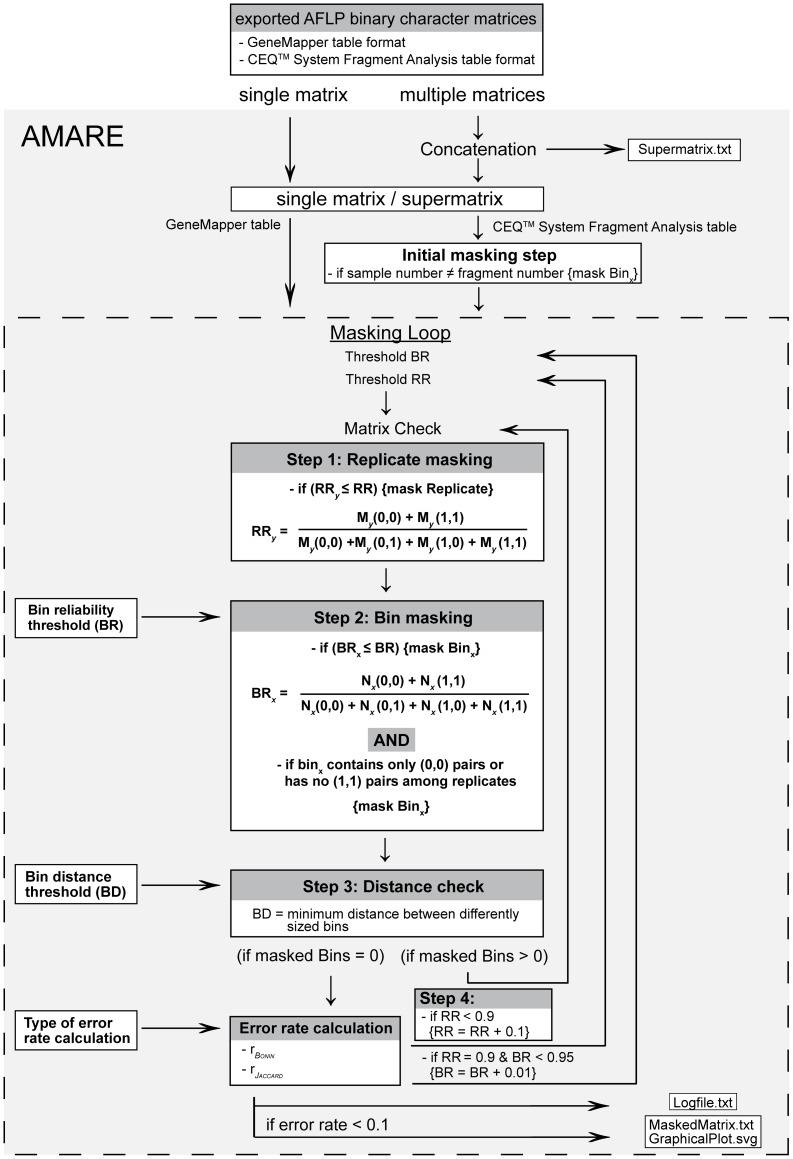
Flow chart of AMARE.

The approach of bin masking among replicates can be separated into four blocks (Figure 1):

- Step 1: First, Replicate reliability, RR_y,_ is checked and replicates are masked, if RR_y_≤RR is true.
- Step 2: Secondly, all bins with BR*_x_*≤BR and without any congruent pairs (1,1) among replicates are masked. AMARE masks all bins without any (1,1) pairs among replicates to avoid spurious background noise in the data. If a bin displays only (0,0) pairs among replicates it is masked as well (by default), because shared fragment absences (null alleles) are particularly prone to homoplasy due to the multiple and independent ways in which a fragment can be lost [3], [6].
- Step 3: The third step consists of a distance check, where all bins which have bin distances less or equal to the allowed BD between differently sized bins are permanently masked. If bins are masked in step 3, RR_y_ for each single replicate potentially changes again. Thus, the process loops back to step 1 maintaining replicate and bin masking achieved in the first round of step 1 and 2. The process will iterate through step 1, 2 and 3 until no further bin or replicate are masked. Replicate mismatch error rates are then calculated for the character matrix (see Figure 1). If the mismatch error rate (r*_Bonin_* or r*_Jaccard_*) <0.1 and the number of remaining bins >5, AMARE generates a new character matrix for the complete data. Predefined thresholds of BR = 0.95 and RR = 0.95 are most likely too conservative [31] and will lead to an excessive masking of bins and loss of signal in the complete data matrix. We therefore decided to use increments of BR and RR to search for matrices with replicate mismatch error rates (r*_Bonin_* or r*_Jaccard_*) <0.1 and a minimum bin number >5.
- Step 4: In step 4, RR is incremented by 0.1 from 0 to 0.9. For each RR threshold, steps 1, 2 and 3 are repeated generating potentially different output matrices.

After the execution of these four steps, BR is incremented by 0.01 starting from a user defined minimal threshold until BR = 0.95. For each BR, steps 1, 2, 3 and 4 are again executed (Figure 1). Thus, for example, if BR was initially set to 0.7, the process potentially generates 26×10 different output matrices. The approach records all matrices with an error rate (r*_Bonin_* or r*_Jaccard_*) <0.1. However, for empirical data we observed that only 4–10 matrices are in fact generated with (r*_Bonin_* or r*_Jaccard_*) <0.1 and a minimum bin number >5. The user might most likely choose the largest (*n′*×*m′*)-character matrix where *n′* is the number of replicates and *m′* is the number of bins after masking.

### Implementation

AMARE is written in Perl. A Perl interpreter must be present in order to execute the software. AMARE requires a preliminary step for bin definition and peak height detection using commercial genotyping software. As input file, AMARE reads exported AFLP binary character matrices in either GeneMapper (Applied Biosystems) or CEQ™ System Fragment Analysis v. 9.0.25 (Beckman Coulter) table format. A mixture of both formats is not possible. The input file contains the AFLP profiles of all sampled individuals including replicates. The subsequent analysis of bin reliability, replicate reliability and error rate estimation is only based on included replicates. AMARE includes three main matrix reduction steps for both formats, plus one initial masking step for the CEQ™ System Fragment Analysis v. 9.0.25 (Beckman Coulter) table format. This table format supplies detailed information on the number of “Fragments” and “Samples” for each individual bin. A difference in the number of “Fragments” and “Samples” means that some samples have two markers (double peaks) within one bin. AMARE masks the bin in the whole character matrix due to the sizing inaccuracy of markers within this bin. Masked bins of the initial masking step are not further considered in the subsequent analysis process.

AMARE concatenates multiple AFLP matrices and then analyzes the concatenated supermatrix in one process. Missing sample data in single matrices are replaced by “?”, which are not further considered in the analyses.

For each individual threshold set, AMARE generates i) a single log file reporting the masking of bins and replicates and ii) a character matrix in text (.txt) and nexus (.nex) format, if error rate conditions and minimum number of remaining bins are met. A summary of all threshold sets and corresponding error rates are stored in the main log file. Nexus files can be directly executed in other programs like PAUP. Additionally, AMARE plots a graphical overview of the original and each masked replicate matrix. Several options can be specified by the user:

1. Concatenation of multiple AFLP character matrices
2. Bin distance threshold, BD
3. Minimum bin reliability threshold, BR
4. Replicate error rate calculation (r*_Bonin_* or r*_Jaccard_*)

Despite the implementation in Perl, AMARE runs very fast. It analyzes 200 different sets of threshold for large data sets with more than 50 replicates and more than 500 bins in less than 10 seconds on a normal desktop computer. AMARE is freely available from http://software.zfmk.de.

### Performance on Real Data

In general the true tree (i.e. species phylogeny) is not known and optimal thresholds for AFLP marker selection may be tested either within well-defined expectations or simulation-based approaches. However, simulations such as AFLP in silico are currently not feasible, as factors influencing bin width and peak height are still poorly understood to be simulated accurately [31].

In the present study the performance of AMARE was tested with empirical, already published AFLP data sets [8], [11], [31] spanning different levels of taxonomic divergence. To decide between different AMARE generated character matrices, we compared corresponding AMARE topologies to phylogenetic results of the original AFLP data sets and/or other independent sources such as morphological and geographical data. For each original and AMARE generated character matrix, we further assessed the effect of marker selection on mismatch error rates (r*_Bonin_* and r*_Jaccard_*), principal coordinate analyses (PCoA), stemminess and resolution scores. Mismatch error rates (r*_Bonin_* and r*_Jaccard_*) were estimated with AMARE, whereas PCoA was calculated using FAMD [36]. A perl script was written to calculate the stemminess value of each resulting tree topology. Stemminess is a tree shape parameter and is defined as the proportion of the sum of internal branch lengths over the total sum of branch lengths of the tree [35]. A low stemminess value indicates a star-like tree, whereas a higher value suggests a more tree-like topology. To calculate the resolution score, we performed 1000 bootstrap replicates for each character matrix. According to Holland et al. [31], all the bootstrap scores over 50% were summed and then divided by the maximum number of internal branches in each tree to give a value between 0% and 100%.

#### Data set of dasmahapatra et al. [8]

Originally, AFLP profiles were visualized by autoradiography and scored by eye. The authors excluded samples with odd AFLP profiles, where most bands were not observed in other profiles and only used consistently amplified loci with sharp bands and minimal size variation. Nineteen replicates of individuals representing five species were generated for all primer combinations to assess the replicate mismatch error rate, r*_Bonin_*.

In the present study, we used the complete AFLP binary 0/1 character matrix as provided by the authors. AMARE matrices were obtained by setting the minimum BR threshold to 0.7 and the BD threshold to 0.0 (because there was no information on fragment length sizes). Both, error rates (r*_Bonin_* and r*_Jaccard_*) were calculated. Phylogenetic analysis were carried out in PAUP* v4.0b10 [37] using neighbor-joining (NJ) on Nei-Li [38] distances corresponding to the analyses of Dasmahapatra et al. [8]. Internal node support was assessed by nonparametric bootstrapping (1000 replicates). Nei-Li distances were used for resolution score, principal coordinate and stemminess analyses.

#### Data set of bonin et al. [11]

Originally, AFLP profiles were visualized and scored by eye with GeneScan Analysis v. 3.7 (Applied Biosystem) and Genographer v. 1.6.0 (http://hordeum.oscs.montana.edu/genographer/). Basically, all markers with a lower peak intensity of 10% of the highest peak’s intensity and loci with less than 3% of band absence for all individuals were excluded from the data set. The authors generated 23 replicates of individuals for five out of ten primer combinations to estimate the replicate mismatch error rate, r*_Bonin_*.

As AMARE is based on replicates, we could only use the five primer combinations with replicates. AMARE matrices were obtained by setting the minimum BR threshold to 0.7 and the BD threshold to 0.15 according to the standard deviation of the sequencer`s sizing precision (ABI Prism 3100 DNA sequencer, Applied Biosystems). Both replicate error rates (r*_Bonin_* and r*_Jaccard_*) were calculated. Phylogenetic analyses were carried out with PAUP* v4.0b10 [37] using NJ on Nei-Li [38] distances corresponding to the original study of Bonin et al. [11]. We analyzed the AFLP binary 0/1 character matrix including all ten primer combinations as provided by the authors, the AFLP binary 0/1 character matrix including the five primer combinations with replicates and the AMARE generated data matrix. Internal node support was assessed by nonparametric bootstrapping (1000 replicates). Nei-Li distances were used for resolution score, principal coordinate and stemminess analyses.

#### Data set of holland et al. [31]

The authors automatically scored the AFLP profiles of the *Ourisia* and the *Ipomoea* data set with GeneMapper v. 3.7 (Applied Biosystems). Optimal parameter settings in GeneMapper for the two data sets were peak height threshold (PHT) 50, minimum fragment length (MFL) 50 (*Ipomoea*) and 100 (*Ourisia*), and bin width (BW) 0.5. As measure of data quality, both replicate mismatch error rates (r*_Bonin_* and r*_Jaccard_*) were calculated for each data matrix. The *Ourisia* data set contained six and the *Ipomoea* data set five replicates of individuals for all primer combinations.

In the present study, the AFLP profiles (ABI.fsa files) as provided by the authors were automatically scored with GeneMapper v.4.1 (Applied Biosystems). At first, we used the optimal parameter settings as described by the authors to get a comparable data matrix. For our own data matrix generation, however, we chose the parameter settings PHT 50, MFL 50 (*Ipomoea*) and 50 (*Ourisia*) and BW 0.85. Subsequently, AMARE was used as a second filter. The BR threshold was set to 0.7 and the BD threshold to 0.15 according to the standard deviation of the sequencer`s sizing precision (3730 Genetic Analyzer, Applied Biosystems). Both error rates (r*_Bonin_* and r*_Jaccard_*) were calculated. Phylogenetic analyses were carried out in PAUP* v4.0b10 [37] using NJ on uncorrected distances corresponding to Holland et al. [31] analyses. Internal node support was assessed by nonparametric bootstrapping (1000 replicates). Uncorrected distances were used for resolution score, principal coordinate and stemminess analyses.

## Results

### Dasmahapatra et al. [8]

The authors genotyped 109 specimens from 23 pinniped species and two outgroup species for 310 AFLP markers with estimated error rates of r*_Bonin_* = 0.004 and r*_Jaccard_* = 0.019. The resolution score of the original character matrix was 46%, the percentage of variation explained by the first three axes of the PCoA 87%, and stemminess calculations of the resulting topology gave a value of 0.85 (Table 2a).

**Table 2:** Overview of character matrices and quality estimates.

a) Dasmahapatra et al. [8] *										
Matrix	BR	RR	# oftaxa	# of replicates	# of markers	resolution score	PCoA^▪^	Error rate (r*_BONIN_*)	Error rate (r*_JACCARD_*)	stemminess
*Original*	*–*	*–*	*109*	*19*	*310*	*45.59*	*86.51*	*0.00400*	*0.01900*	*0.850662887*
**1a**	**0.70–0.78**	**0.0–0.9**	**109**	**19**	**108**	**28.06**	**91.65**	**0.00877**	**0.01432**	**0.977431345**
2a	0.79–0.89	0.0–0.9	109	19	107	27.96	91.64	0.00689	0.01118	0.977357985
3a	0.90–0.94	0.0–0.9	109	19	102	26.42	92.65	0.00206	0.00346	0.977265256
4a	0.95	0.0–0.9	109	19	98	26.72	93.44	0.00000	0.00000	0.980829109
**b) Bonin et al.** [**11**] *										
**Matrix**	**BR**	**RR**	**# of** **taxa**	**# of replicates**	**# of markers**	**resolution score**	**PCoA^▪^**	**Error rate (r** ***_BONIN_*** **)**	**Error rate (r** ***_JACCARD_*** **)**	**stemminess**
*Original*	*–*	*–*	*189*	*23*	*222*	*10.69*	*26.44*	*0.03400*	*0.10400*	*0.11268627*
1b	0.70–0.78	0.0–0.8	189	23	177	10.09	51.87	0.02653	0.06742	0.118347772
2b	0.79–0.82	0.0–0.8	189	23	176	09.65	52.02	0.02544	0.06462	0.115813324
3b	0.83–0.86	0.0–0.8	189	23	175	09.60	52.09	0.02460	0.06234	0.117876953
4b	0.87–0.91	0.0–0.8	189	23	170	09.94	51.84	0.02148	0.05508	0.121583951
5b	0.92–0.95	0.0–0.8	189	23	151	06.95	50.31	0.01325	0.03469	0.123875627
6b	0.70–0.77	0.9	188	22	177	09.77	52.08	0.02260	0.05714	0.115907542
7b	0.78–0.86	0.9	188	22	176	09.96	52.23	0.02144	0.05418	0.115681322
8b	0.87–0.90	0.9	188	22	173	08.52	52.07	0.01944	0.04930	0.120184544
**9b**	**0.91–0.95**	**0.9**	**188**	**22**	**158**	**07.67**	**52.19**	**0.01266**	**0.03223**	**0.125378018**
**c) Holland et al.** [**31**] **: ** ***Ourisia*** **^♦^**										
**Matrix**	**BR**	**RR**	**# of** **taxa**	**# of replicates**	**# of markers**	**resolution score**	**PCoA^▪^**	**Error rate (r** ***_BONIN_*** **)**	**Error rate (r** ***_JACCARD_*** **)**	**stemminess**
*Original*	*–*	*–*	*24*	*6*	*2011*	*66.55*	*22.38*	*0.143*	*0.531*	*0.088816069*
**1c**	**0.70–0.83**	**0.0–0.7**	**24**	**6**	**530**	**40.73**	**24.72**	**0.080**	**0.163**	**0.112753464**
2c	0.84–0.95	0.0–0.7	24	6	286	38.16	26.20	0.000	0.000	0.117756188
3c	0.70–0.74	0.8	22	4	515	47.06	26.36	0.090	0.153	0.11130232
4c	0.75–0.95	0.8	22	4	340	45.77	26.90	0.000	0.000	0.123701587
**d) Holland et al.** [**31**] **: ** ***Ipomoea*** **^♦^**										
**Matrix**	**BR**	**RR**	**# of** **taxa**	**# of replicates**	**# of markers**	**resolution score**	**PCoA^▪^**	**Error rate (r** ***_BONIN_*** **)**	**Error rate (r** ***_JACCARD_*** **)**	**stemminess**
*Original*	*–*	*–*	*25*	*5*	*1425*	*68.02*	*33.77*	*0.163*	*0.490*	*0.129984309*
**1d**	**0.70–0.79**	**0.0–0.7**	**25**	**5**	**406**	**42.03**	**38.15**	**0.090**	**0.172**	**0.189308632**
2d	0.80–0.95	0.0–0.7	25	5	216	36.97	40.13	0.000	0.000	0.223652373
3d	0.70–0.74	0.8	24	4	411	51.74	38.76	0.090	0.148	0.216727081
4d	0.75–0.95	0.8	24	4	269	43.12	42.64	0.000	0.000	0.254480608

Selected character matrices are indicated in **bold**, original character matrices in *italics*.

* = Nei-Li distances were used for resolution score, principal coordinate and stemminess analyses.

♦ = Uncorrected distances were used for resolution score, principal coordinate and stemminess analyses.

▪ = percentage of variation explained by the first three axes of the principle coordinate analyses (PCoA).

**Figure 2:**
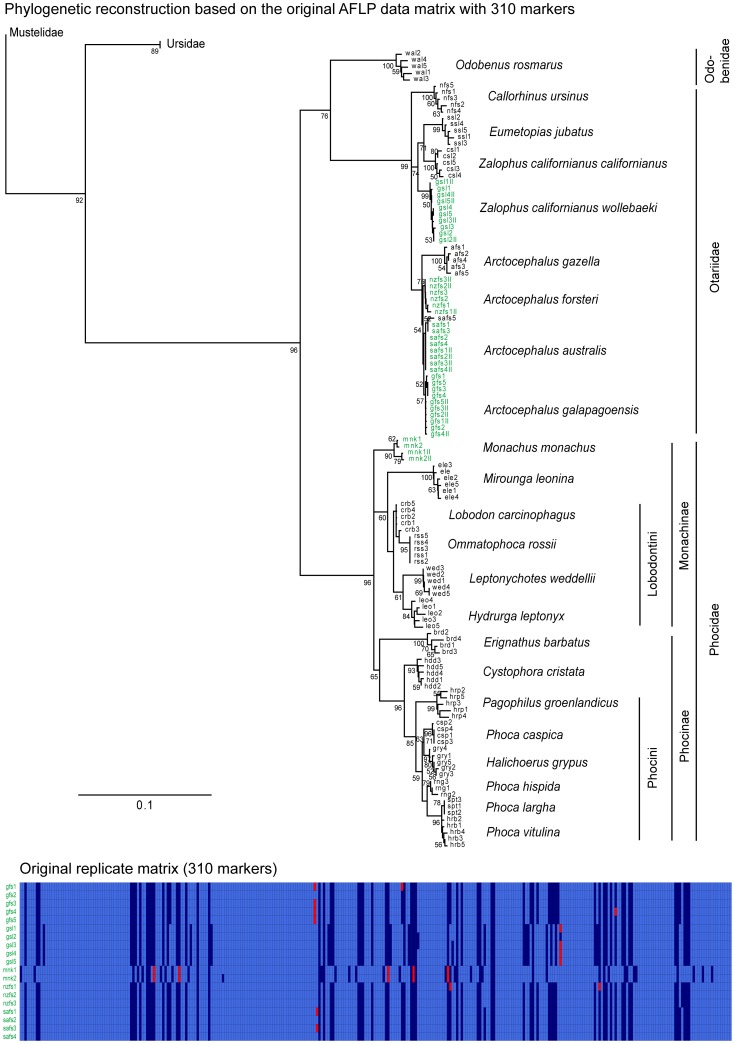
Rooted neighbor-joining tree of the original character matrix of Dasmahapatra et al. [8]. The original character matrix comprised 310 selected markers. The tree is based on Nei-Li genetic distances. Numbers represent % bootstrap support obtained from 1000 bootstrap replicates and are shown only when ≥50%. Replicated individuals are indicated in green. A graphical overview of the replicates character matrix is shown below. In this matrix each row represents a replicate pair of a single individual and each column a bin. Light blue cells specify reproducible (0,0) bin states, dark blue cells reproducible (1,1) bin states and red cells unreproducible (0,1) bin states. Scale bar indicates Nei-Li distance.

**Figure 3:**
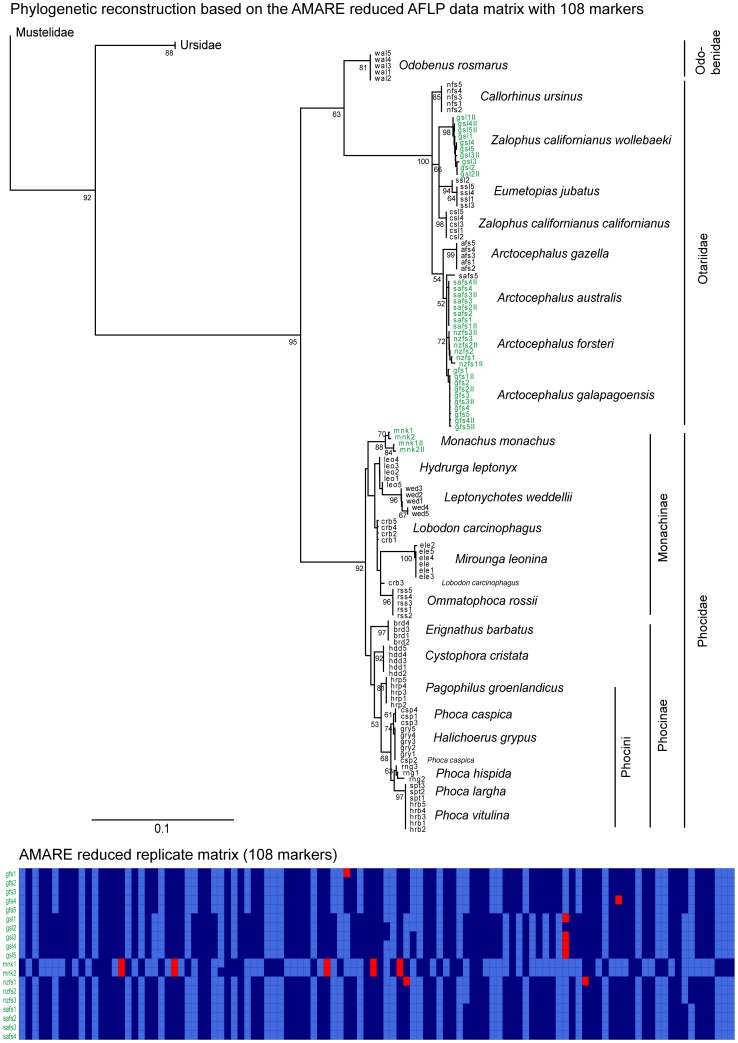
Rooted neighbor-joining tree of the AMARE reduced character matrix of Dasmahapatra et al. [8]. The AMARE reduced character matrix comprised 108 selected markers. The tree is based on Nei-Li genetic distances. Numbers represent % bootstrap support obtained from 1000 bootstrap replicates and are shown only when ≥50%. Replicated individuals are indicated in green. A graphical overview of the replicates character matrix is shown below. In this matrix each row represents a replicate pair of a single individual and each column a bin. Light blue cells specify reproducible (0,0) bin states, dark blue cells reproducible (1,1) bin states and red cells unreproducible (0,1) bin states. Scale bar indicates Nei-Li distance.

As described above, AMARE gradually increases the user specified minimum BR threshold until BR = 0.95 and generates character matrices for each individual threshold set. Altogether, AMARE generated four different character matrices for the pinniped data set. Compared to the original character matrix, the resolution score decreased for all four AMARE matrices. PCoA and stemminess values, however, increased for the four matrices. Compared to the original pinniped matrix, the error rate (r*_Bonin_*) increased for matrix 1a and matrix 2a, but decreased for matrix 3a and 4a (Table 2a). Among the four AMARE matrices, we chose character matrix 1a, although it did not consistently give the best results over all the quality estimations (resolution score = 28%; PCoA = 92%; stemminess = 0.98) (Table 2a). The resulting phylogeny, however, was most similar to the topology based on the original pinniped data set (Figures 2, 3). Character matrix 1a consisted of 108 selected markers and was generated by the following parameter settings of AMARE: BR = 0.7–0.78, RR = 0.0–0.9 and BD = 0.0. The estimated replicate mismatch error rates were r*_Bonin_* = 0.009 and r*_Jaccard_* = 0.014.

The NJ tree was rooted with *Meles meles* (Mustelidae). The trees of the original and of the AMARE masked character matrix were topologically almost identical and had only minor differences in branch support (Figures 2, 3). The main differences concerned the position of *M. leonina* and the monophyly of two species, *L. carcinophagus* and *P. caspica*. In the NJ tree of the original AFLP matrix, *M. leonina* was placed as sister group to the Lobodontini and *L. carcinophagus* as well as *P. caspica* each formed monophyletic groups (Figure 2). In contrast, *M. leonina* was sister to *O. rossii* and neither *L. carcinophagus* nor *P. caspica* were monophyletic in the AMARE NJ tree (Figure 3). Comparing original and AMARE masked replicate data matrices, the proportion of invariant (0,0) bins accounted for 200 markers in the original character matrix (Figures 2, 3).

### Bonin et al. [11]

The authors scored 190 individuals of the common frog (*Rana temporaria*) for 328 AFLP markers. Replicates of individuals, however, were generated for only five primer combinations. The estimated mismatch error rates of this matrix were r*_Bonin_* = 0.034 and r*_Jaccard_* = 0.104 for 222 scored AFLP markers and 189 individuals. Resolution score and PCoA analyses gave 11% and 26%, respectively. The stemminess value of the tree was 0.113 (Table 2b).

Nine distinct character matrices were generated by AMARE. Compared to the original character matrix, stemminess and PCoA values increased, whereby error rates (r*_Bonin_* and r*_Jaccard_*) decreased for each AMARE generated matrix. All AMARE matrices had a lower resolution score than the original matrix (Table 2b).

**Figure 4:**
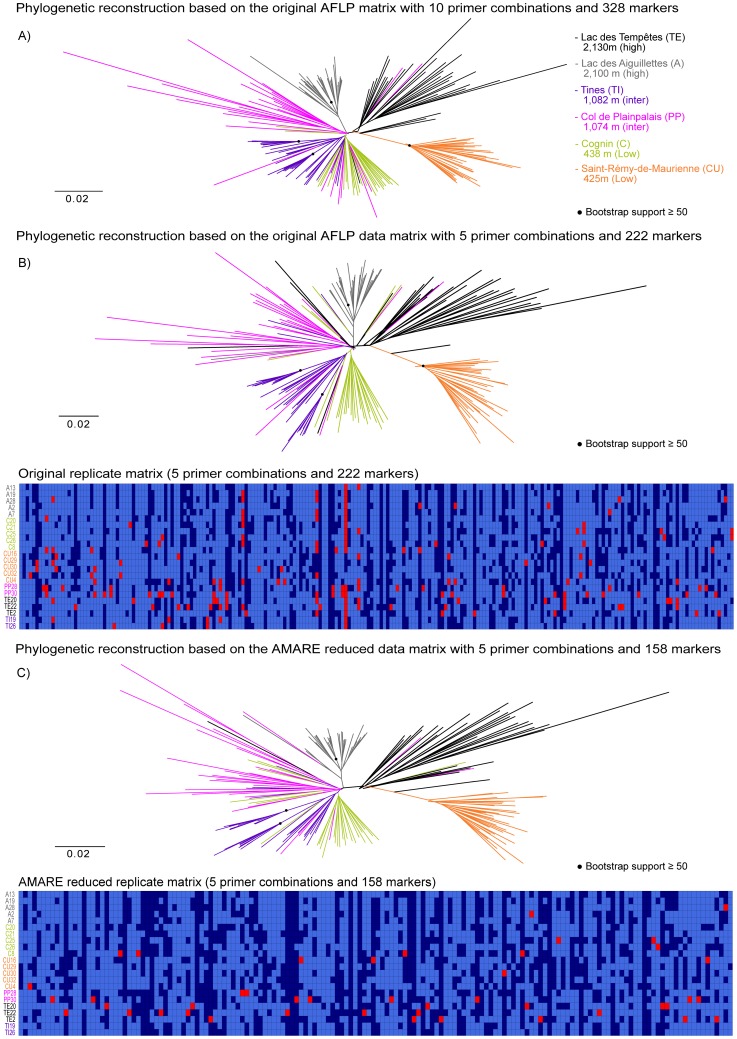
Data set of Bonin et al. [11]. The unrooted neighbor-joining trees are based on Nei-Li genetic distances calculated from A) the orignal character matrix with 328 markers (10 primer combinations), B) the original character matrix with 222 markers (the 5 primer combinations including replicates) and C) the AMARE masked character matrix with 158 markers. Bootstrap support values ≥50% (1000 bootstrap replicates) are labeled with • and are only shown for more basal splits. Each sample site is indicated by its own colour. A graphical overview of the replicates character matrix is shown below. In this matrix each row represents a replicate pair of a single individual and each column a bin. Light blue cells specify reproducible (0,0) bin states, dark blue cells reproducible (1,1) bin states and red cells unreproducible (0,1) bin states. Scale bar indicates Nei-Li distance.

We selected character matrix 9b with 158 markers and estimated error rates of r*_Bonin_* = 0.013 and r*_Jaccard_* = 0.032. The underlying parameter settings of AMARE were BR = 0.91–0.95, RR = 0.9 and BD = 0.15. Due to the strict RR threshold, replicates of the individual TE1 were excluded from the data set. The resolution score of matrix 9b was 7.7%, the PCoA value 52% and the stemminess 0.13. Phylogenetic reconstructions based on matrix 9b gave best results grouping common frog individuals according to sample sites (Figure 4).

The phylogenetic reconstructions of the original AFLP data matrices were based on ten (328 markers) and five primer combinations (222 markers) and showed no major changes compared to the phylogenetic reconstruction based on the AMARE masked matrix (Figure 4). Individuals of Saint-Rémy-de-Maurienne formed a monophyletic group in all NJ trees and were closely related to a clade mainly comprising individuals of Lac des Tempêtes. In the tree based on the AMARE generated matrix, both populations were genetically more differentiated from all other populations than in the original trees. Individuals of Lac des Aiguillettes were monophyletic only in the NJ tree of the original AFLP matrix with 10 primer combinations. Bootstrap support values were similar between trees based on different character matrices, though there was no bootstrap support ≥50 for the monophyly of the population of Saint-Rémy-de-Maurienne in the AMARE tree.

### Holland et al. [31]

The resulting binary character matrices generated by GeneMapper v. 4.1 comprised 2011 (*Ourisia*) and 1425 (*Ipomoea*) selected markers. The *Ourisia* data set contained 24 specimens representing 13 species and the *I. batatas* data set 25 specimens including *I. tiliacea* as outgroup species. The estimated error rates of the *Ourisia* character matrix were r*_Bonin_* = 0.143 and r*_Jaccard_* = 0.531 and of the *Ipomoea* character matrix r*_Bonin_* = 0.163 and r*_Jaccard_* = 0.49. The resolution score of the original *Ourisia* matrix was 67%, the PCoA yielded 22% and the stemminess value was 0.089. The original *Ipomoea* matrix had a resolution score of 68%, a PCoA value of 34% and a stemminess value of 0.13 (Table 2c and d).

**Figure 5:**
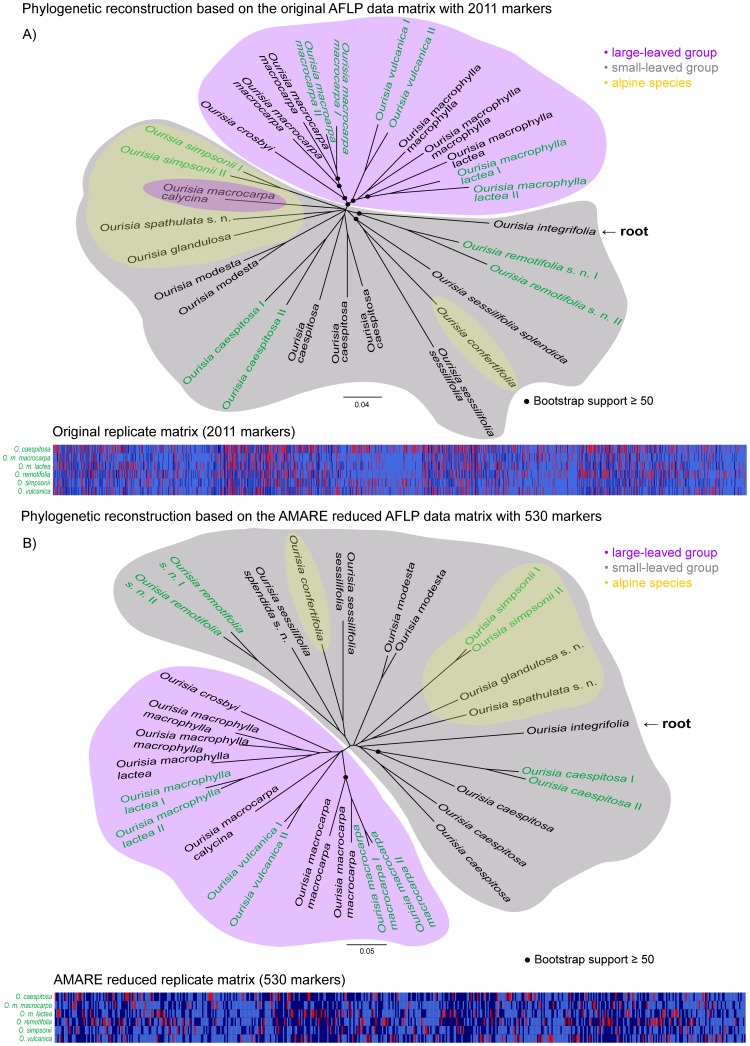
*Ourisia* data set of Holland et al.[31]. The neighbor-joining trees are based on uncorrected genetic distances calculated from A) the original character matrix with 2011 markers and B) the AMARE masked character matrix with 530 markers. Bootstrap support values ≥50% (1000 bootstrap replicates) are labeled with • and are only shown for more basal splits. Replicated individuals are indicated in green. The Australian species *Ourisia integrifolia* is sister to all New Zealand species and thus the root of the tree. A graphical overview of the replicates character matrix is shown below. In this matrix each row represents a replicate pair of a single individual and each column a bin. Light blue cells specify reproducible (0,0) bin states, dark blue cells reproducible (1,1) bin states and red cells unreproducible (0,1) bin states. Scale bar indicates uncorrected genetic distance.

AMARE generated four different character matrices for the *Ourisia* data set. Whereas PCoA and stemminess values increased for all four AMARE matrices, resolution scores and error rates decreased compared to the original character matrix (Table 2c). Among AMARE generated matrices, we selected the character matrix 1c with 530 markers and estimated error rates of r*_Bonin_* = 0.08 and r*_Jaccard_* = 0.163. The resolution score and PCoA value of the matrix were 41% and 22%, respectively. Stemminess calculations yielded a value of 0.11. The AMARE parameter settings of matrix 1c were as followed: BR = 0.7–0.83, RR = 0.0–0.7 and BD = 0.15. Though the tree topology of the AMARE masked matrix was partly different from that of the original *Ourisia* data set, it corresponded to results based on a much larger *Ourisia* data set published by Meudt et al. [19]. According to the results of the stemminess calculations, the AMARE NJ tree was more tree-like than the NJ tree of the original character matrix (Figure 5).

Basically, the AMARE NJ tree resolved two clades, one comprising all large-leaved and the other comprising all small-leaved *Ourisia* species. Additionally, a lineage including the three alpine species *O. simpsonii, O. glandulosa* and *O. spathulata* was identified within the small-leaved group. In the phylogeny based on the original character matrix not all large-leaved species grouped together, and *O. m. calycina* was found in the small-leaved group as sister group to *O. simpsonii*. In general bootstrap support was lower in the AMARE phylogenetic tree. Matrix size was reduced from 2011 to 530 markers (Figure 5).

**Figure 6:**
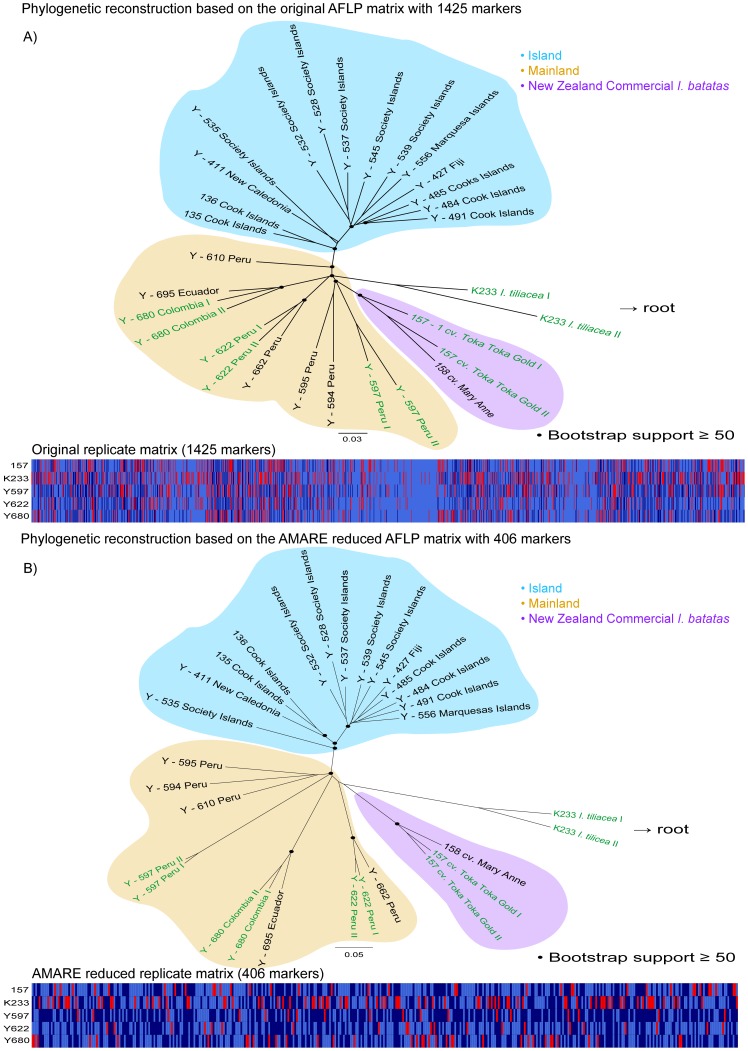
*Ipomoea* data set of Holland et al. [31]. The neighbor-joining trees are based on uncorrected genetic distances calculated from A) the original character matrix with 1425 markers and B) the AMARE masked character matrix with 406 markers. Bootstrap support values ≥50% (1000 bootstrap replicates) are labeled with •. Replicated individuals are indicated in green. *Ipomoea tiliacea* represents the root of the tree. A graphical overview of the replicates character matrix is shown below. In this matrix each row represents a replicate pair of a single individual and each column a bin. Light blue cells specify reproducible (0,0) bin states, dark blue cells reproducible (1,1) bin states and red cells unreproducible (0,1) bin states. Scale bar indicates uncorrected genetic distance.

AMARE generated four different character matrices for the *Ipomoea* data set. PCoA and stemminess values increased for the four AMARE matrices, whereas resolution scores and error rates decreased in comparison to the original *Ipomoea* character matrix (Table 2d). We chose the character matrix 1d with 406 markers for further analysis based on the following parameter settings: BR = 0.7–0.79, RR = 0.0–0.7 and BD = 0.15. The estimated was r*_Bonin_* = 0.09 and r*_Jaccard_* = 0.172, the resolution score was 42%, the PCoA yielded 38% and the stemminess value was 0.19. The topologies of both phylogenetic trees were very similar and specimens of *I. batatas* were separated into a mainland (South America) and an island clade (Figure 6). Whereas the New Zealand Commercial *I. batatas* “Mary Anne” and “Toka Toka Gold” were sister to all other *I. batatas* in the AMARE NJ tree, they showed a sister group relationship to *I. batatas* from Peru in the tree of the original data matrix. According to stemminess calculations, the AMARE NJ phylogeny was more tree-like than the NJ phylogeny based on the original matrix. Bootstrap support in the AMARE phylogeny, however, were lower than in the original tree. A comparison of the replicate matrices (Figure 6) showed a decrease in the number of selected markers from 1425 to 406.

## Discussion

### Optimality Criteria

Holland et al. [31] demonstrated that strict masking of unreliable bins optimizing error rates among replicates might become counter productive in phylogenetics due to the considerable loss of valuable characters. Instead, they proposed to select character matrices with maximal resolution scores accepting a certain amount of unreliable bins. In the present study, we tested the performance of AMARE with empirical data sets and assessed the effect of marker selection on error rate estimations [10], resolution scores [31], PCoA [28] and stemminess [35] calculations. We showed that AMARE increases the PCoA and stemminess values of AFLP character matrices, and reduces mismatch error rates and resolution scores (Table 2). The reduction of resolution scores seems surprising at first hand, but can be explained by the fact that characters with exclusive (0,0) bin states among replicates are masked in the complete data. This masking leads to a loss of conflict-free fragment absence (0) characters in the masked matrix and thus reduces the resolution score. Null alleles (i.e. fragment absences) are certainly less reliable characters than fragment presence (1) characters [3], [6]. We therefore consider high resolution scores of unmasked character matrices as potentially inflated scores. AMARE generates multiple masked character matrices. It turns out that in three out of four tested data sets (Table 2: 1a, 1c, and 1d) the selection of masked character matrices with highest resolution scores gave best results. In case of the *Ourisia* data set [31], the selected AMARE matrix (1c) even gave a topologically more congruent phylogenetic result than the original matrix, when compared with morphological and molecular data of a much larger data set of the genus *Ourisia* [19]. PCoA and stemminess values increased for AMARE generated matrices compared to the original data sets, suggesting that both quality estimates represent adequate optimality criteria for matrix selection (Table 2). Among the different AMARE masked character matrices, phylogenetic reconstructions based on matrices with highest PCoA and stemminess values were less resolved than AMARE matrices with lower PCoA and stemminess values (not shown). Equally, AMARE masked matrices with lowest error rates yielded poorly resolved phylogenies, possibly as too many characters have been masked to support robust tree reconstructions.

### Empirical Data

The re-analyses of published empirical data corroborates the usefulness of the AMARE approach. Both data sets of Bonin et al. [11] and Dasmahapatra et al. [8] were manually scored. In each case, phylogenetic reconstructions based on the original data matrices were similar in topology to those based on selected AMARE matrices (Figures 2, 3, 4). The pinniped data set [8] was extremely reduced from 310 to 108 selected markers. A comparison of the pinniped replicate matrices (Figures 2, 3) showed that mainly markers with invariant (0,0) bin states were masked in the original character matrix. The performance of AMARE depends on a representative sample of replicates. In case of the pinniped data set replicates were not representative for the whole data set (see Figures 2, 3) and marker selection was just based on the genetic diversity of the family Otariidae. The family Phocidae was only represented by the replicates of the species *M*.* monachus*. In fact, topological differences between the AMARE tree and the original phylogeny were especially found in the Phocidae clade. Bootstrap support values ≥50 decreased in the AMARE tree compared to the original pinniped tree. This resulted most likely from a strongly reduced number of markers and the reduction of conflict-free (0,0) bin states which increased bootstrap support in the original pinniped tree. A comparison of the error rate (r*_Bonin_*) between the original and AMARE masked character matrix showed that the lower error rate (r*_Bonin_*) of the original pinniped data matrix was mainly due to the high amount of (0,0) bin states lowering the apparent error rate [31].

The AMARE masked replicate matrix of the common frog data set [11] showed an relative increase of phylogenetic valuable (1,1) bin states and a decline of erroneous (1,0) bin states (Figure 4). In the frog data set replicates represented the genetic diversity of the whole data set. In this example, however, we could only use five out of ten primer combinations due to the limited generation of replicates. Probably the use of all ten primer combinations would have resulted in a phylogenetic tree with a more robust grouping of individuals according to sample sites.

Both, the *Ourisia* and the *Ipomoea* data set [31] were automatically scored with commercial available scoring software. In each case, phylogenetic reconstructions based on the AMARE masked data matrices were more tree-like than the phylogenies based on the original data sets (Figures 5, 6). For data matrix generation, we decided to use a different bin width (BW) definition than those recommended by Holland et al. [31]. We considered the recommended BW of 0.5 too small, splitting identical alleles into separate characters [9]. Instead, we chose a BW of 0.85 considering the standard deviation of 0.15 of the sequencer`s sizing precision (3730 Genetic Analyzer, Applied Biosystems) to prevent a merging of adjacent but separate markers into one character.

The number of selected markers extremely decreased from 2011 to 530 markers in the AMARE masked *Ourisia* character matrix. The phylogenetic tree clearly resolved a large-leaved and a small-leaved group within the genus (Figure 5). This result corresponded to the result based on a much larger *Ourisia* data set published by Meudt et al. [19]. Furthermore, within the small-leaved group a monophyletic lineage with the three alpine species *O. simpsonii, O. glandulosa* and *O. spathulata* was found. The data of Meudt et al. [19] also supported a monophyletic alpine group within the small-leaved clade though including *O. confertifolia, O. glandulosa* and *O. spathulata*. Meudt et al. [19] argue, however, that *O. simpsonii* could be also included in this alpine group based on morphological characters. In the AMARE tree *O. confertifolia* did not group within the alpine lineage, but was sister to *O. s. sessilifolia*.

The AMARE phylogeny of the *Ipomoea* data set was more tree-like than the original *Ipomoea* tree and showed a higher genetic differentiation into a mainland and an island group (Figure 6). Replicates always grouped as sister taxa in the AMARE phylogeny, but not in the original *Ipomoea* tree. This is due to the fact that AMARE selects markers dependent on the marker reproducibility among replicates. Replicates were representative in the *Ourisia* data set, but not in the *Ipomoea* data set. In the *Ipomoea* example, genetic diversity was only represented by the replicates of the mainland group. Generally, the number of replicates was very low in both data sets. The original and AMARE masked replicate matrices of both data sets were not directly comparable because different BWs were used to generate respective matrices. Generally, error rates were quite high due to the small number of replicates [31]. Bootstrap support values ≥50 strongly decreased in the AMARE NJ trees. Not only the number of markers was much higher in the original AFLP data matrix but also the small BW of 0.5 could have artificially increased bootstrap values by splitting one character into two eventually doubling bootstrap support values in the original phylogeny.

### Conclusions

The application of AFLPs has demonstrated its merits in population genetics and phylogenetics. However, due to the nature of the AFLP technique the assessment of marker homology and reliability has been an issue since the introduction of this method. Several attempts have been published which have been designed to increase scoring reliability particularly in population genetics. These attempts address automated scoring of AFLP profiles concerning peak height and bin width [9], [26], [28], [29], [31]. A special issue of concern has been the lack of a reliable and fully automated control of marker reproducibility itself.

In this study, we showed that the AFLP scoring process can be fully automated and issues of bin width, peak height, and reproducibility can be addressed in a combined approach using commercial software packages and AMARE. AMARE implements characteristics of manually scoring processes in an objective, fast, and perfectly reproducible way and goes beyond published efforts in population genetics and phylogenetics relying on AFLP technique. We showed that making marker selection dependent on marker reproducibility improved the signal-to-noise ratio of AFLP character matrices. The ease with which enormous amounts of AFLP data can be generated makes automated scoring inevitable.
